# The Significant Morbidity and Mortality Indicators in Patients of Cirrhosis

**DOI:** 10.7759/cureus.21226

**Published:** 2022-01-14

**Authors:** Manaswinee Mallik, Abhishek Singhai, Sagar Khadanga, Vaibhav Ingle

**Affiliations:** 1 Medicine, All India Institute of Medical Sciences, Bhopal, Bhopal, IND; 2 Internal Medicine, All India Institute of Medical Sciences, Bhopal, Bhopal, IND; 3 General Medicine, All India Institute of Medical Sciences, Bhopal, Bhopal, IND

**Keywords:** meld score, child-pugh score, mortality, morbidity, cirrhosis

## Abstract

Background: Cirrhosis progression varies greatly from patient to patient due to a variety of factors, including hepatic reserve, cirrhosis etiology, and the presence of hepatocellular cancer. As a result, determining a prognosis in a patient with cirrhosis remains a difficult task. For nearly three decades, the Child-Pugh score (CPS) has been the gold standard for determining the prognosis of cirrhosis. In the last two decades, many prognostic models and scores like a model for end-stage liver disease (MELD), chronic liver failure-sequential organ failure assessment (CLIF-SOFA) score, peripheral blood lymphocyte to monocyte ratio (LMR) have been presented to predict prognosis in patients with cirrhosis and to choose the best therapy option. The aim of our study is to determine which score is more effective in predicting three-month mortality and whether these scores are equally effective in predicting short-term outcomes.

Materials & methods: In this hospital-based longitudinal study, we analyzed 140 patients with cirrhosis of liver visiting All India Institute of Medical Sciences Bhopal between July 2019 and July 2020. All the 140 patients were followed up for three months to establish short-term outcomes. The blood investigations were done at the time of presentation from all the patients and after three months in the survivors. Various scores were calculated.

Results: The majority of patients (47%) were in Child-Pugh class C. Mean MELD score was 13.54, LMR score was 1.96 and CLIF-SOFA score was 5. The total bilirubin, serum creatinine, international normalized ratio (INR), total leukocyte count, absolute monocyte count, CPS, MELD, CLIF-SOFA were significantly higher in a non-surviving group as compared to the surviving group, whereas the albumin and LMR significantly decreased in the non-surviving group. On performing multivariate regression, LMR and CLIF-SOFA were significant independent risk factors of mortality after adjusting for confounding factors. All the parameters had significant discriminatory power to predict mortality. Discriminatory power of CLIF-SOFA (AUC 0.808; 95% CI: 0.733 to 0.870) was excellent and discriminatory power of CPS (AUC 0.792; 95% CI: 0.716 to 0.856), MELD score (AUC 0.765; 95% CI: 0.685 to 0.832) and LMR (AUC 0.75; 95% CI: 0.669 to 0.819) was acceptable. Among all the parameters, CLIF-SOFA was the best predictor of mortality at a cut-off point of >5 with 80.80% chances of correctly predicting mortality.

Conclusion: The significant morbidity and mortality indicators are high total bilirubin, high creatinine, high INR, high TLC, low platelet count, and low albumin. Among the various scores, CLIF-SOFA is a better predictor of mortality and morbidity. Low LMR and high CLIF-SOFA are significant independent risk factors of mortality at three months.

## Introduction

Cirrhosis of the liver progresses over time, resulting in end-stage liver disease which is irreversible [1,2]. However, when the underlying etiology is curable, it is reversible. It has been predicted that Cirrhosis of the liver would affect more than 50 million people worldwide by 2025. According to the Centre for Disease Control and Prevention's National Vital Statistics Report 2017, nearly four million people in the United States had cirrhosis, accounting for 1.8 % of the adult population. The liver disease accounts for approximately two million deaths per year worldwide. Cirrhosis is currently the 11th most common cause of death globally [3]. Cirrhosis claimed the lives of 41,473 people (12.8 per 100,000) in the year 2017 [4]. It is one of the major causes of increased mortality and morbidity worldwide as well as in India with a prevalence of 4.5% to 9% [5]. The cirrhosis of the liver has diverse etiologies, viruses, NASH, and alcohol being commonest [6].

The Child-Pugh score (CPS) has been the gold standard for determining cirrhosis prognosis for nearly three decades. It was initially used to predict mortality during surgery as well as to determine prognosis and the need for a liver transplant. Points 5-6, classified as CPS class A, have a one-year survival rate of 100% and a two-year survival rate of 85%. Points 7-9, classified as CPS B, have a one-year survival rate of 80% and a two-year survival rate of 60%. Points 10-15, classified as CPS C, have a one-year survival rate of 45% and a two-year survival rate of 35%. The score includes five measures total bilirubin, serum albumin, prothrombin time/international normalized ratio (PT/INR), ascites, and hepatic encephalopathy (HE) [7].

Another scoring system used in cirrhosis of the liver is called the model for end-stage liver disease (MELD) score. Initially, it was used for predicting mortality in three months of surgery in patients who had undergone a transjugular intrahepatic portosystemic shunts (TIPS) procedure and used for determining prognosis and prioritizing for receipt of a liver transplant, with the highest risk of death will receive the highest preference for organ donation. The MELD score was found to be a good predictor of mortality in patients with end-stage liver disease. This score shifted the organ allocation policy, ensuring that not only “patients come first,” but also “the sickest patient comes first. MELD score includes three objective variables as serum bilirubin, INR for PT, and serum creatinine. There is a European study that says the MELD score is an excellent predictor of both short and medium-term survival, and an increase in MELD score is associated with a decrease in residual liver function. It is calculated by the formula “MELD score = 6.43+3.78 Ln(serum total bilirubin[mg/dL])+11.2 Ln(INR) +9.57Ln(serum creatinine[mg/dL].” Despite the fact that the MELD score is the nearest to the ideal score, it has some flaws, such as its inaccuracy in predicting survival in 15%-20% of cases [8,9].

The imbalance between prooxidants and antioxidant potential, known as oxidative stress, plays a key role in the progression of inflammatory, metabolic, and proliferative chronic liver disease. It is one of the major causes of sudden deterioration as well as the sudden death of liver cirrhosis patients [10,11]. Inflammation and immune deficiency are responsible for 30% of mortality in cirrhosis [12]. Monocytes are important cells in the pathogenesis of liver fibrosis. The dysfunction of B lymphocytes is seen in their memory cell and there is a reduction in helper and cytotoxic T-lymphocytes [13,14]. The importance of hematological markers of inflammation from complete blood counts has become the subject of research. One of the well-known inflammatory markers is the lymphocyte to monocyte ratio (LMR). It can be used to know the utility of this marker in predicting the outcome in cirrhosis of the liver [15].

The CLIF-SOFA score was created to assess acute cirrhosis decompensation. Acute decompensation is described by the emergence of a severe complication such as HE, ascites, gastrointestinal bleeding, or bacterial infection within a short period of time. The CLIF-SOFA score was adjusted from the SOFA score by evaluating the liver (bilirubin level), cerebral function (HE grade), coagulation (INR, circulation (mean arterial pressure), and lung (PaO_2_/FiO_2_ or SpO_2_/FiO_2_) systems [16]. A CLIF-SOFA score of 6 indicates a higher six-week mortality rate, while a CLIF-SOFA score of 7 indicates a higher hospital mortality rate. The CLIF-SOFA scores can be easily determined and used to predict prognosis [17]. ACLF diagnostic criteria are based on analyses of patients with organ failure as defined by the CLIF-SOFA score, which helps in predicting mortality in patients with ACLF [18]. There are studies where the CLIF-SOFA was compared with the current models. One study for patients with alcoholic liver cirrhosis validated scoring systems. Another study found that CLIF-SOFA was more accurate than other scoring methods in predicting four-week death [19]. The SOFA has been a major determinant in 30-day mortality in another study [16].

CPS and MELD scores have widely been used to predict cirrhotic patient outcomes. They do, however, have some disadvantages. First, two of the CPS components (ascites and HE) are subjective and may vary depending on the physician's assessment and the use of diuretics and lactulose. Second, in liver cirrhosis, INR, which is a component of both the CPS and MELD scores, does not adequately reflect coagulopathy and, as a result, liver function [20]. Third, the INR value varies from one laboratory to the next [21].

The relationship among the parameters CPS, MELD, CLIF-SOFA, LMR has not been studied yet. In our study, we are aimed to assess the better score in predicting three-month mortality. We hypothesize that low LMR and high CLIF-SOFA are associated with increased mortality in cirrhosis of the liver of all etiologies and it is equally efficacious as MELD and CPS in predicting mortality.

## Materials and methods

We conducted a hospital-based longitudinal study with an intended follow-up duration of three months in tertiary care hospital, All India Institute of Medical Sciences (AIIMS), Bhopal between July 2019 and July 2020. This study was approved by Institutional Ethical Committee (No. IHEC-LOP/2019/MD0089). The written informed consent was taken from the study population. Those patients who agreed for the participation were included in this study. There were given patient information sheet both in English as well as Hind for their ease.

The target population included all adults above 18 years of age with cirrhosis screened from the medicine ward of AIIMS, Bhopal. The criteria for exclusion included age younger than 18 years, presence of malignancy such as hepatocellular carcinoma, hematological malignancy, colorectal cancer, nasopharyngeal carcinoma, pancreatic cancer, lymphoma, autoimmune diseases, chronic infection like tuberculosis, HIV, presence of sepsis, and patients who have received antibiotics in last 14 days. All the 140 patients were followed up for three months to establish short-term outcomes.

The blood sample was drawn at the time of admission from all the patients and after three months in the survivors. Following investigations done: complete blood count, total protein, albumin, total bilirubin, alanine aminotransferase, aspartate aminotransferase, creatinine, prothrombin time (PT), INR, arterial blood gas analysis. Various scores were calculated based on these investigations. CPS, CLIF-SOFA points were calculated by adding points manually, and peripheral blood LMR was calculated manually from the complete blood count report whereas MELD was calculated by the standard formula by the electronic calculator Cleveni inv version 0.8.1 [1]. Patients were followed up after three months both in person as well as telephonically. LMR and CLIF-SOFA scores were analyzed extensively as compared to the pre-existing CPS and MELD, to find out its short-term mortality outcomes.

Statistical analysis

The data entry was done in the Microsoft EXCEL spreadsheet and the final analysis was done with the use of Statistical Package for Social Sciences software ver 21.0 (IBM, Chicago, IL, USA). The categorical variables were presented as number and percentage (%), the quantitative data were presented as the means ± SD and median with 25th and 75th percentiles (interquartile range). The data normality was checked by using the Kolmogorov-Smirnov test. Paired t-test/Wilcoxon signed-rank test was used for comparison across follow-up. We constructed the receiver operating characteristic (ROC) curve for CPS, MELD score, LMR, and CLIF-SOFA scores and estimated the area under the curve (AUCs) for each of these scores for three-month mortality. ROC curve was used to determine the overall predictiveness of CPS, MELD, LMR, and CLIF-SOFA. Sensitivity, specificity, positive predictive value (PPV), and negative predictive value (NPV) were calculated. The cut-off value for CPS, MELD, LMR and CLIF-SOFA are >B, >20.49, <1.66, and >5, respectively. For statistical significance, a p-value of less than 0.05 was considered significant.

## Results

Total 140 patients of cirrhosis of the liver were included in the study between July 2019 and August 2020. All the 140 patients were followed up for three months to establish short-term outcomes. The basic characteristics of study subjects are listed in Table 1. The mean age of the patients was 47 (+13) years and 106 (75.7%) were male. Alcohol was the most common etiology of cirrhosis. The majority of patients (47%) were in Child-Pugh class C. Mean MELD score was 15.88±9.48 with a median of 13.54 (8.718-22.033), mean LMR score was 2.72±2.6 with a median of 1.96 (1.198-3.028) and mean value of CLIF-SOFA score was 5.56±3.91 with a median 5 (3-7).

**Table 1:** Characteristics of the study population, difference between the variables among non-survivors and survivors patients *Independent t-test, ‡ Fisher's exact test, § Chi-square test, **Mann Whitney U test NASH - nonalcoholic steatohepatitis; INR - international normalized ratio; MELD score - model for end-stage liver disease score; LMR - lymphocyte to monocyte ratio; CLIF SOFA - chronic liver failure-sequential organ failure assessment

Variables	Study subject (n=140)	Non-surviving (n=65)	Surviving (n=75)	P-value
Female/male	34 (24.29%)/106 (75.71%)	12 (35.29%)/53 (50%)	22 (64.71%)/53 (50%)	0.135^§^
Age (years) (Mean+SD)	47.75± 13	47.91 ± 12.89	47.61 ± 13.18	0.894^*^
Illiterate/literate	108 (77.14%)/32 (22.86%)	13 (40.63%)/52 (48.15%)	19 (59%)/56 (51.85%)	0.454^§^
Etiology				
Alcohol-related	64 (45.71%)	33 (51.56%)	31 (48.44%)	0.548^‡^
Hepatitis B	24 (17.14%)	9 (37.50%)	15 (62.50%)	
Hepatitis C	11 (7.86%)	7 (63.64%)	4 (36.36%)	
NASH	12 (8.57%)	4 (33.33%)	8 (66.67%)	
Cardiac	9 (6.43%)	5 (55.56%)	4 (44.44%)	
Budd chiarisyndrome	3 (2.14%)	1 (33.33%)	2 (66.67%)	
Biliary	4 (2.86%)	1 (25%)	3 (75%)	
Autoimmune	6 (4.29%)	3 (50%)	3 (50%)	
Wilson disease	1 (0.71%)	1 (100%)	0 (0%)	
Cryptogenic	6 (4.29%)	1 (16.67%)	5 (83.33%)	
Duration since diagnosis (months) (Median)	6 (0-12) months	6 (0-24)	6 (0-12)	0.621**
Total protein (g/dL) (mean±SD)	6.18 ± 0.95	6.04 ± 0.92	6.3 ± 0.97	0.116*
Albumin (g/dL) (Median)	2.51 (2.078-2.907)	2.2 (1.95-2.59)	2.82 (2.195-3.2)	<0.0001>
Total bilirubin (mg/dL) (Median)	2.08 (1.008-5.098)	4.12 (1.76-8.32)	1.36 (0.89-2.675)	<0.0001>
Creatinine (mg/dL) (Median)	1.02 (0.8-1.592)	1.22 (0.89-2.01)	0.9 (0.725-1.26)	0.002**
INR (Median)	1.46 (1.218-1.79)	1.67 (1.39-2.3)	1.3 (1.15-1.535)	<0.0001>
Total leukocyte count (10^3cells/mL) (Median)	7.4 (4.995-10.212)	8.02 (6.31-10.9)	6.62 (4.725-9.795)	0.005**
Platelets (Median) (10^3cells/mL)	116 (68.75-177.75)	104 (65-175)	120 (73-180.5)	0.516**
Absolute monocyte count 10^3cells/mL) (Median)	0.53 (0.314-0.9)	0.69 (0.46-1.09)	0.4 (0.251-0.7)	<0.0001>
Absolute lymphocyte count (10^3cells/mL)(Median)	1.12 (0.77-1.702)	1.04 (0.782-1.52)	1.15 (0.72-1.775)	0.638**
Child-Pugh class (Median)	9 (7-11)	11 (9-12)	8 (7-9)	<0.0001>
Child-Pugh class A	13 (9.29%)			
Child-Pugh class B	66 (47.14%)			
Child-Pugh class C	61 (43.57%)			
MELD score (Median)	13.54 (8.718-22.033)	21.03 (12.17-27.42)	10.36(7.4-14.6)	<0.0001>
LMR score (Median)	1.96 (1.198-3.028)	1.38 (0.93-2.26)	2.57 (1.73-4.325)	<0.0001>
CLIF-SOFA score (Median)	5 (3-7)	3 (2-5)	7 (5-10)	<0.0001>

There was no significant difference in relation to age, gender, education, underlying etiology, duration since diagnosis, total protein, absolute lymphocyte count, and platelet counts between the two groups. The total bilirubin, serum creatinine, INR, total leukocyte count, absolute monocyte count (AML), CPS, MELD, CLIF-SOFA were significantly elevated in the non-surviving group as compared to the surviving group, whereas the albumin and LMR were significantly lower in a non-surviving group. On performing multivariate regression, LMR and CLIF-SOFA were significant independent risk factors of mortality after adjusting for confounding factors. With the higher LMR, the risk of mortality is significantly lower with an odds ratio of 0.634 (0.456 to 0.882). With the high CLIF-SOFA, the risk of mortality is significantly high with an odds ratio of 1.34 (1.013 to 1.773). All the parameters had similar discriminatory power to predict mortality.

**Figure 1:**
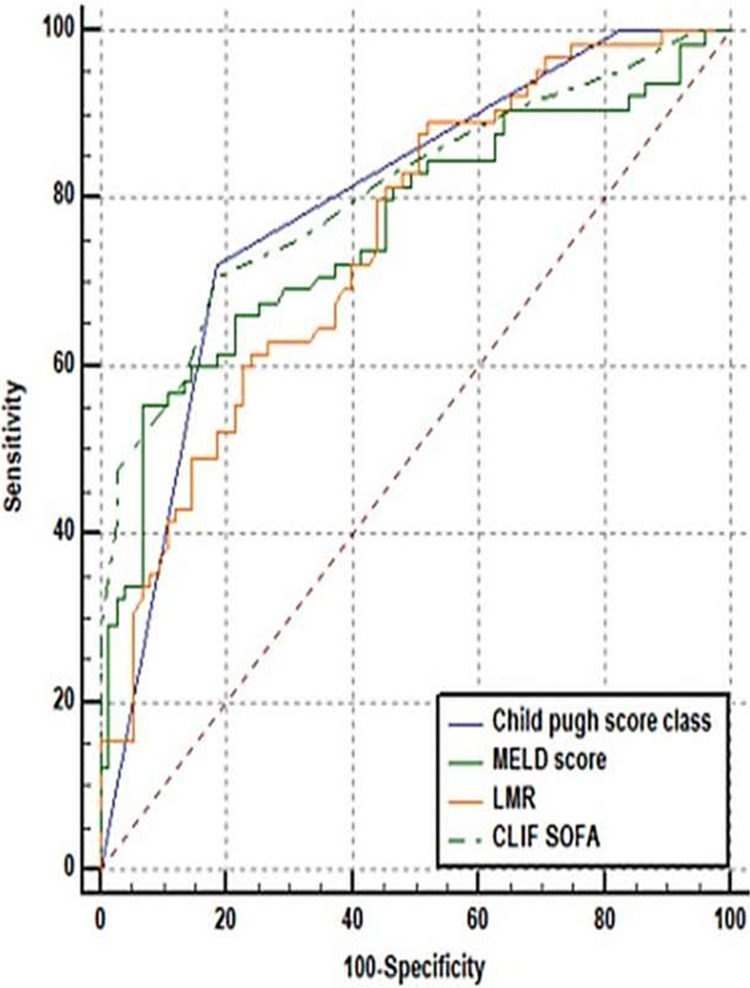
Receiver operating curve of various prediction scores for three months mortality as an outcome MELD score - model for end-stage liver disease score; LMR - lymphocyte to monocyte ratio; CLIF SOFA - chronic liver failure-sequential organ failure assessment

The CLIF-SOFA, CPS, MELD, and LMR scores have similar AUCs of 0.808, 0.792, 0.765, and 0.75, respectively. Among all the parameters, CLIF-SOFA was the best predictor of mortality at the cut-off point of >5 with 80.80% chances of correctly predicting mortality. CPS had a sensitivity of 72.31%, on the other hand, the MELD score had a specificity of 93.33%. In the prediction of mortality, LMR had the lowest specificity of 76.00%. The highest PPV was found in the MELD score (87.80%) and the highest NPV was found in CPS (77.20%). It is shown in Table 2. No significant difference was seen in an AUC of CPS, MELD score, LMR, and CLIF-SOFA to predict mortality (Figure 1, Table 3).

**Table 2:** Receiver operating characteristic curve of CPS, MELD, LMR, and CLIF-SOFA score to predict mortality ROC - Receiver operating curve; PPV - positive predictive value; NPV - negative predictive value

Mortality	Child-Pugh score (CPS) class	Model for end-stage liver disease (MELD) score	Lymphocyte to Monocyte Ratio (LMR)	Chronic liver failure-sequential organ failure assessment (CLIF-SOFA)
Area under the ROC curve (AUC)	0.792	0.765	0.75	0.808
Standard Error	0.0338	0.0417	0.0407	0.0373
95% Confidence interval	0.716 to 0.856	0.685 to 0.832	0.669 to 0.819	0.733 to 0.870
P-value	<0.0001	<0.0001	<0.0001	<0.0001
Cut off	>B	>20.49	≤1.66	>5
Sensitivity (95% CI)	72.31% (59.8%-82.7%)	55.38% (42.5%-67.7%)	61.54% (48.6%-73.3%)	70.77% (58.2%-81.4%)
Specificity (95% CI)	81.33% (70.7%-89.4%)	93.33% (85.1%-97.8%)	76% (64.7%-85.1%)	81.33% (70.7%-89.4%)
PPV (95% CI)	77% (64.5%-86.8%)	87.8% (73.8%-95.9%)	69% (55.5%-80.5%)	76.7% (64.0%-86.6%)
NPV (95% CI)	77.2% (66.4%-85.9%)	70.7% (60.7%-79.4%)	69.5% (58.4%-79.2%)	76.2% (65.4%-85.1%)
Diagnostic accuracy	77.14%	75.71%	69.29%	76.43%

 

**Table 3:** Difference in variables between non-surviving and surviving patients *Independent t-test, ‡ Fisher's exact test, § Chi-square test, **Mann Whitney U test NASH - nonalcoholic steatohepatitis; INR - international normalized ratio; CPS - Child-Pugh score; MELD score - model for end-stage liver disease score; LMR - lymphocyte to monocyte ratio; CLIF SOFA - chronic liver failure-sequential organ failure assessment

Variables	Non-surviving (n=65)	Surviving (n=75)	P-value
Female/male	12/53	22/53	0.135^§^
Ag e(years)	47.91 ± 12.89	47.61 ± 13.18	0.894^*^
Illiterate /literate	13/52	19/56	0.454^§^
Etiology			
Alcohol-related	33	31	0.548^‡^
Hepatitis B	9	15	
Hepatitis C	7	4	
NASH	4	8	
Cardiac	5	4	
Budd Chiari syndrome	1	2	
Biliary	1	3	
Autoimmune	3	3	
Wilson disease	1	0	
Cryptogenic	1	5	
Duration since diagnosis (months)	6 (0-24)	6 (0-12)	0.621**
Total protein (g/dL)	6.04 ± 0.92	6.3 ± 0.97	0.116*
Albumin (g/dL)	2.2 (1.95-2.59)	2.82 (2.195-3.2)	<0.0001>
Total bilirubin (mg/dL)	4.12 (1.76-8.32)	1.36 (0.89-2.675)	<0.0001>
Creatinine (mg/dL)	1.22 (0.89-2.01)	0.9 (0.725-1.26)	0.002**
INR	1.67 (1.39-2.3)	1.3 (1.15-1.535)	<0.0001>
Total leukocyte count (10^3^cells/mL)	8.02 (6.31-10.9)	6.62 (4.725-9.795)	0.005**
Platelets (10^3^cells/mL)	104 (65-175)	120 (73-180.5)	0.516**
Absolute monocyte count (10^3^cells/mL)	0.69 (0.46-1.09)	0.4 (0.251-0.7)	<0.0001>
Absolute lymphocyte count (10^3^cells/mL)	1.04 (0.782-1.52)	1.15 (0.72-1.775)	0.638**
CPS	11 (9-12)	8 (7-9)	<0.0001>
MELD	21.03 (12.17-27.42)	10.36 (7.4-14.6)	<0.0001>
LMR	1.38 (0.93-2.26)	2.57 (1.73-4.325)	<0.0001>
CLIF-SOFA	3 (2-5)	7 (5-10)	<0.0001>

## Discussion

In our study, out of the 140 patients with cirrhosis of liver included in the study between July 2019 and August 2020, 61 (47%) were in Child-Pugh class C, 75 patients survived while 65 patients did not survive at the end of three months. The most common etiology of cirrhosis was alcohol in our study. The total bilirubin, serum creatinine, INR, total leukocyte count, AML, CPS, MELD, and CLIF-SOFA was significantly higher in the non-surviving group as compared to a surviving group, whereas the LMR score is significantly higher in surviving group (p-value < 0.0001 in all). The LMR and CLIF-SOFA are significant independent risk factors of mortality. With higher LMR, the risk of mortality is significantly lower whereas, with higher CLIF SOFA, the mortality is significantly higher (odds ratio 1.34).

We can routinely use the LMR score in our day-to-day practice which can be easily achievable from a single peripheral complete blood count. It is inexpensive compared to other scores. As CLIF-SOFA score is a better predictor of short-term mortality which was applied for acute decompensation of cirrhosis, a score of 6 indicates a higher six-week mortality rate, while 7 indicates a higher hospital mortality rate. In our study also, we found higher CLIF-SOFA, having a significantly higher three-month mortality rate. Among the four variables CLIF-SOFA was the best predictor of mortality at a cut-off point of >5 with 80.80% chances of correctly predicting mortality, the CLIF-SOFA can be applied to cirrhotic patients who have presented with acute decompensation.

In a study by Zhu et al., the non-surviving group had a substantially lower LMR. A high MELD score and a low LMR were both independent risk factors for three-month mortality in their study [15]. In a retrospective cohort study by Zhang et al., LMR was statistically lower in the non-surviving group and the LMR was closely correlated to the MELD score [22]. In a most recent study which was done in 2019, it was found that lower LMR is associated with increasing one-month mortality among cirrhosis patients [23]. Lower LMR might be due to inflammatory response which occurs in cirrhosis of the liver. Inflammation triggers monocyte release from the bone marrow to the peripheral blood and differentiation of blood monocytes into tissue macrophages. A decrease in peripheral lymphocytes count may be due to increased cell death or migration to the liver due to inflammation [12,24]. The AUC for the MELD, CPS, LMR, and CLIF-SOFA is 0.765, 0.792, 0.75, and 0.808, respectively. In a study by Zhang et al., AUC for MELD score, which was approximately 0.9, and for LMR was 0.8 [22]. In a study by Jamil et al., the AUC of MELD, CPS, and LMR are 0.958, 0.760, and 0.807, respectively [25]. In our study, no significant difference was seen in the AUC of MELD, CPS, LMR, and CLIF SOFA to predict mortality. Though CLIF-SOFA is a better predictor of mortality but not statistically significant as compared to other scores. In a study by Jeong et al., CLIF-SOFA was a significant factor for 30-day mortality [16]. For predicting mortality the cut-off sensitivity and specificity of the MELD score of our study are 20.49, 55.38%, and 93.33%, respectively, while these values are higher in other studies [22]. The cut-off values of sensitivity and specificity of LMR of our study are £1.66, 61.54%, and 76%, respectively whereas in Zhang et al. study, it was 2.10%, 82.6%, 78.8%, respectively [22], and in Jamil et al.'s study, it was £3.31, 80%, and 74.83%, respectively [25].

The strength of our study is that a similar study among the CPS, MELD, LMR, and CLIF-SOFA scores is not available in the literature.

The limitation of our study is being a single-center study with small sample size. Another limitation is that few patients expired outside our hospital, therefore, the cause of death was not clear in some cases. Prospective, multi-center studies are needed to validate further morbidity and mortality indicators in patients with cirrhosis.

## Conclusions

We provided an overview regarding the CPS, MELD, CLIF-SOFA, and LMR scores. All of these scores are having similar mortality predicting outcomes. Low LMR and high CLIF-SOFA are significant independent risk factors of mortality at three months. The easily achievable and inexpensive LMR score can be used in daily practice to predict short-term mortality in cirrhosis patients. But further studies might be required to identify the subjects where we should use CPS, MELD, LMR, CLIF-SOFA scores for assessment of short-term outcomes and the timing when we should use these scores for assessing prognosis. A further multicentric large-scale study is required to confirm our data.
